# Supplementary material for: The causal relationship between the human gut microbiota and pyogenic arthritis: a Mendelian randomization study
Source: Front Cell Infect Microbiol. 2024 Nov 26;14:1452480. doi: 10.3389/fcimb.2024.1452480 (PMC11629706; doi:10.3389/fcimb.2024.1452480)
Supplement: Supplementary file 1 [file DataSheet1.zip › mendelian test/ebi-a-GCST90027782.csv_forest.pdf]

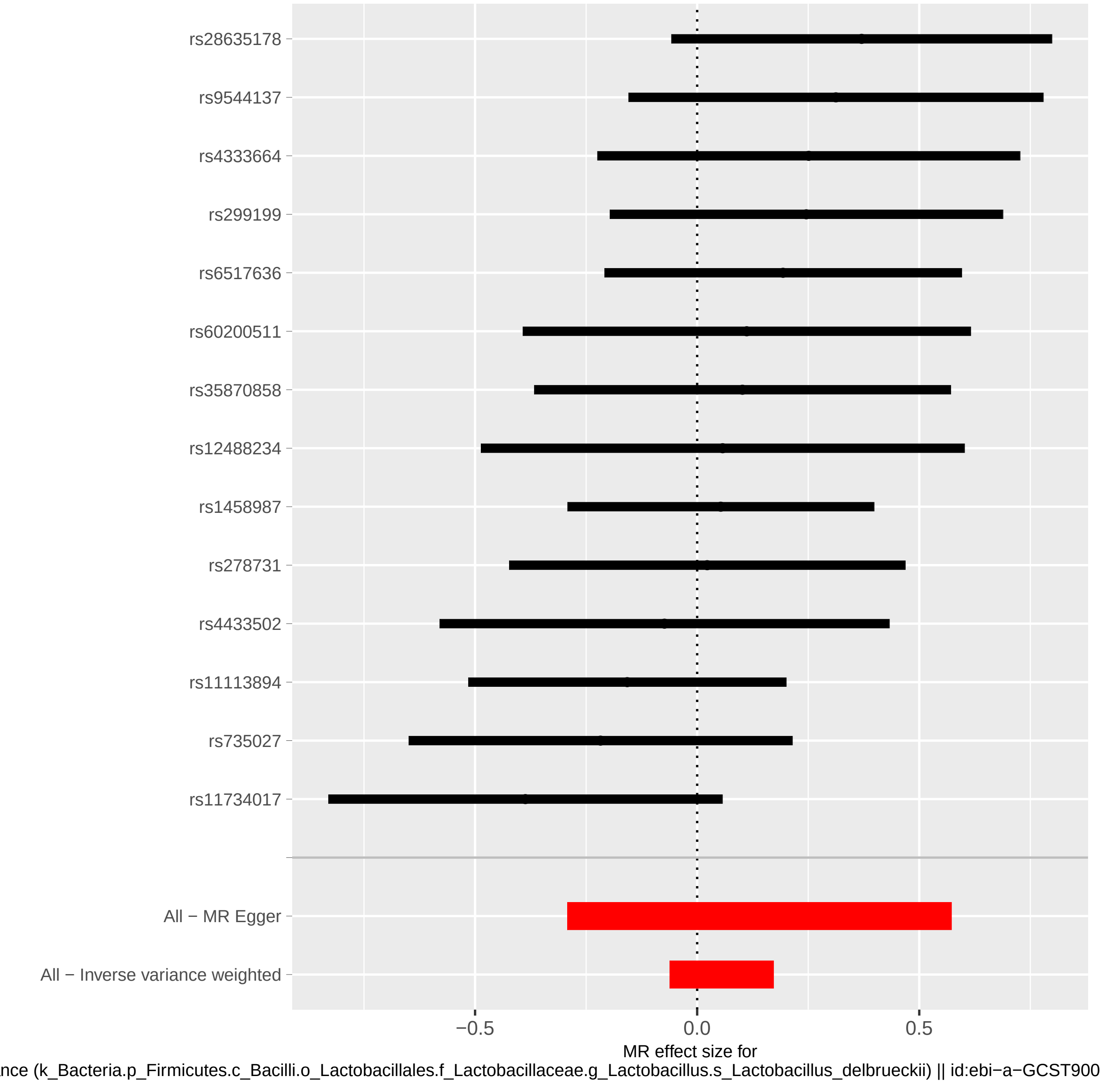
rs28635178
rs9544137
rs4333664
rs299199
rs6517636
rs60200511
rs35870858
rs12488234
rs1458987
rs278731
rs4433502
rs11113894
rs735027
rs11734017
All − MR Egger
All − Inverse variance weighted
−0.5
0.0
0.5
MR effect size for
nce (k_Bacteria.p_Firmicutes.c_Bacilli.o_Lactobacillales.f_Lactobacillaceae.g_Lactobacillus.s_Lactobacillus_delbrueckii) || id:ebi−a−GCST900
